# Acute liver failure precipitated by acute Budd‐Chiari syndrome and complete portal vein thrombosis

**DOI:** 10.1002/ccr3.4326

**Published:** 2021-05-25

**Authors:** Yuji Suzuki, Akiko Suzuki, Keisuke Kakisaka, Yasuhiro Takikawa

**Affiliations:** ^1^ Division of Hepatology Department of Internal Medicine Iwate Medical University School of Medicine Yahaba‐cho Japan

**Keywords:** Acute Budd‐Chiari syndrome, acute liver failure, liver transplantation, portal vein thrombosis

## Abstract

Acute Budd‐Chiari syndrome and complete portal vein thrombosis are important features of myeloproliferative neoplasm that may occur simultaneously, resulting in acute liver failure. Liver transplantation could be effective even in such a catastrophic condition without post‐transplant complications.

## Graphical abstract

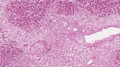

A 46‐year‐old man complaining of fatigue and right upper quadrant pain was referred to our hospital; findings revealed abdominal distention. Contrast‐enhanced computed tomography revealed complete thrombotic occlusion of the main trunk of the portal and splenic veins (Figure 1A, arrows), as well as the three main hepatic veins (Figure 1B, arrows). Laboratory findings revealed leukocytosis (leukocytes, 25 610/μL) and acute liver failure (alanine aminotransferase, 3188 IU/L; aspartate transaminase, 3804 IU/L; total bilirubin, 2.4 mg/dL; ammonia, 140 μg/dL; and international normalized ratio, 2.24). Although low‐molecular‐weight heparin was administered subcutaneously, the patient developed acute liver failure with grade III hepatic encephalopathy 5 days after admission. Ten days after admission, emergency orthotopic liver transplantation was performed. Microscopic examination of the explanted liver showed massive hepatocyte loss in the centrilobular region (Figure 2, hematoxylin and eosin staining, 10×). The patient was eventually diagnosed with JAK2 V617F mutation‐positive myeloproliferative neoplasm and was treated with hydroxyurea and aspirin, following liver transplantation.^1^, ^2^ Over the 5‐year follow‐up period, he remains well without post‐transplant complications.

**FIGURE 1:**
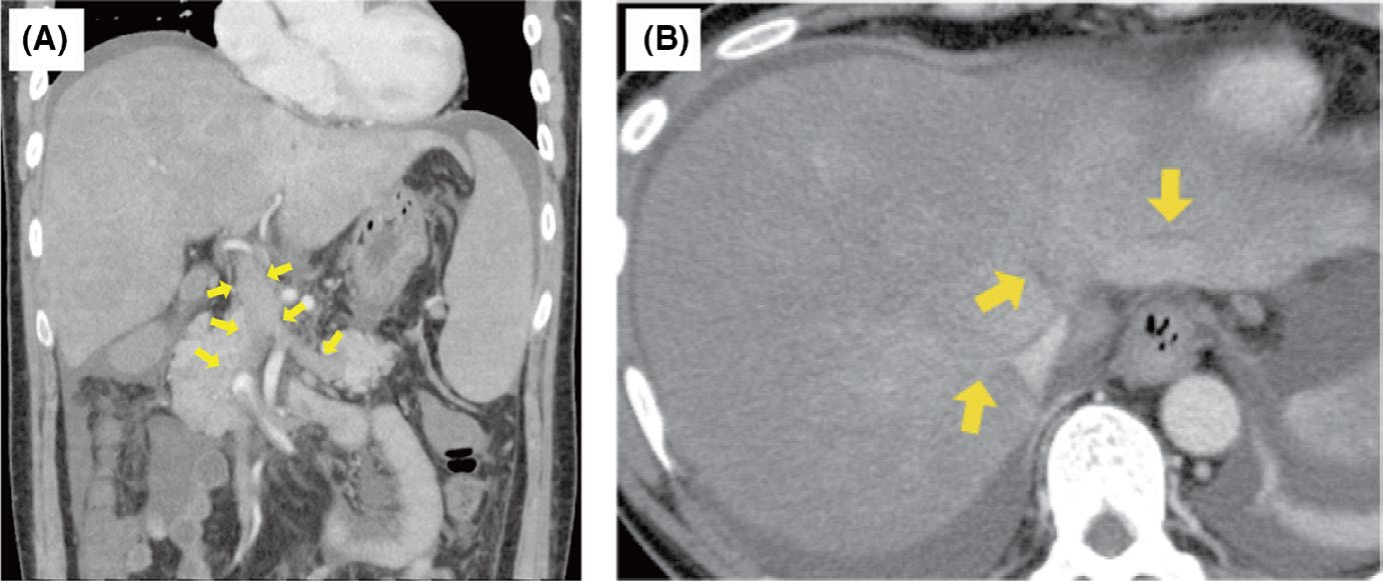
Coronal contrast‐enhanced computed tomography in the portal venous phase shows decreased attenuation in the portal vein and splenic vein without any signs of cavernous transformation. Yellow arrows indicate thrombosis (A). Axial contrast‐enhanced computed tomography in the portal venous phase shows decreased attenuating thrombus in the three main hepatic veins (arrows), compression of inferior vena cava, enlargement of the liver, and presence of ascites (B)

**FIGURE 2:**
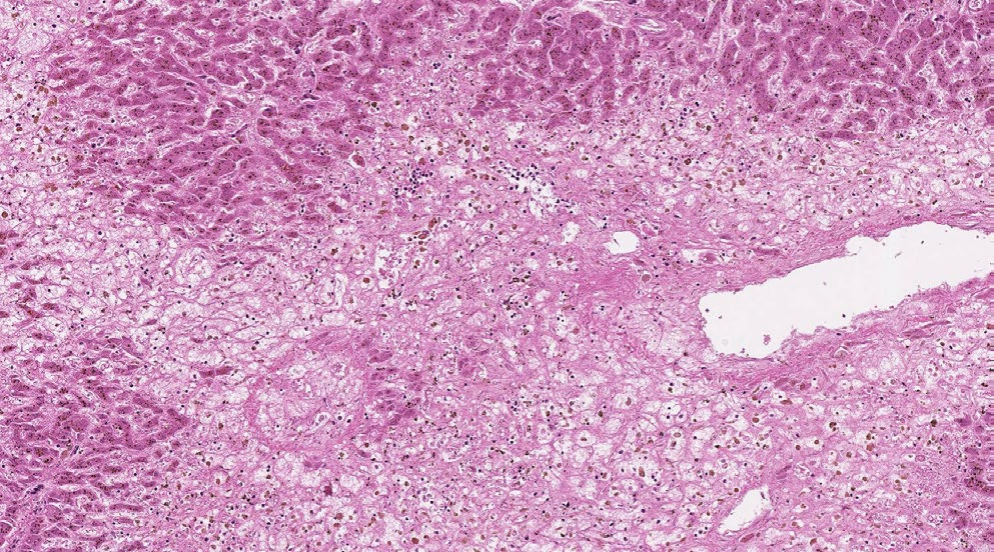
Explanted liver specimen shows massive hepatocyte loss in the centrilobular region (hematoxylin and eosin staining, original magnification ×10)

We demonstrated that acute Budd‐Chiari syndrome and complete portal vein thrombosis might occur simultaneously by myeloproliferative neoplasm, resulting in acute liver failure. Liver transplantation could be effective even in such a catastrophic condition.

## CONFLICT OF INTEREST

The authors declared no potential conflict of interest with respect to the research, authorship, and/or publication of this article.

## AUTHOR CONTRIBUTIONS

YS: wrote the manuscript. AS: contributed to the clinical data collection. KK and YT: supervised the case report.

## ETHICAL APPROVAL

Informed consent was obtained from the patient to publish his case and medical images. No ethical committee approval was required for the publication of this case report.

## Data Availability

The data that support the findings of this study are available on request from the corresponding author. The data are not publicly available due to privacy of the patient.
